# Stress and cataract surgery: A nationwide study evaluating surgeon burnout

**DOI:** 10.1177/11206721231154611

**Published:** 2023-02-03

**Authors:** Abdus Samad Ansari, Annie See Wah Tung, David M Wright, Patrick Watts, Gwyn Samuel Williams

**Affiliations:** 1 Singleton Eye Unit, Sketty Lane, Swansea, UK; 2 Section of Academic Ophthalmology, School of Life Course Sciences, FoLSM, King's College London, London, UK; 3 University Hospital Wales, Cardiff, UK; 4 The Centre for Public Health, School of Medicine, Dentistry & Biomedical Sciences, 1596 Queen's University Belfast, Belfast, UK

**Keywords:** Burnout, cataract surgery, stress, ophthalmology, United Kingdom

## Abstract

**Background:**

We aimed to evaluate the nationwide prevalence of stress induced burnout among cataract surgeons. We believe that knowledge of these factors can help formulate a solution to this underreported problem.

**Methods:**

A three-part nationwide cross-sectional survey was disseminated with via the Royal College of Ophthalmologists (RCOphth) in the United Kingdom(UK). All consultants, trainees and specialty doctors and associate specialists(SAS) were invited to participate. We evaluated burnout using the Maslach Burnout Inventory (MBI). Logistic regression modelling was completed to look at factors linked to high level burnout in certain domains.

**Results:**

A total of 406 respondents completed our survey. Prevalence of cataract surgery-related high burnout was estimated at 3.45% (Section A and/or B) and 40% within Personal Accomplishment (PA)(Section C of the MBI). Multiple factors were associated with increased burnout within PA: Increasing age: 61+ OR: 2.99 (1.02–8.78, *p *=* *0.05), Number of cataract operations completed: >3000 OR 2.98 (1.03–8.64, *p *=* *0.04), Lists per week: 2: OR 2.99 (1.38–6.47, *p *<* *0.01), 2.5: OR 8.95 (2.58–31.02, *p *<* *0.01), 3 or more: OR 2.64 (1.07–6.54, *p *=* *0.04). Sleeping 8 h or more was found to be protective OR 0.52 (0.28–0.96, *p *=* *0.04). 17% of respondents indicated they would be willing to give up cataract surgery if given the opportunity.

**Conclusion:**

The prevalence of stress induced burnout by cataract surgery appears to be present in a minority of surgeons. There appears to be a significant reduction in the feeling of personal achievement within the profession. A large proportion of this cohort appears to be willing to give up surgical responsibilities.

## Introduction

The concept of burnout was first described in the early 1970's by Freudenberger.^1^ He defined this concept as a mental state of fatigue or frustration that originated from professional relationships that failed to produce the desired outcomes or rewards.^1^ Subsequently in 1982 Maslach outlined burnout as a psychological syndrome encompassing emotional exhaustion, depersonalisation and a diminished sense of personal accomplishment that occurred in professionals working with others in challenging environments. His view derived from the theory of burnout itself undermining the care and professional attention given to clients of human service including healthcare professionals and is now accepted to be a repeatable way of measuring stress-related burnout in the medical profession.^2^ Although there is no consensus on the optimal method of measuring burnout, the Maslach Burnout Inventory (MBI) is by far the most commonly used instrument in trying to quantify this.^3^ It comprises three dimensions of burnout; emotional exhaustion (EE), depersonalisation (DP) and personal accomplishment (PA). Other authors have since been able to confirm its utilisation as a tool in research methodology, commending its three dimensionality.^4,5^

Phacoemulsification is one of the most common and successful surgical procedures performed worldwide. Improvements in surgical techniques, anaesthesia and equipment have brought with it a new age of personal, social, and psychological demands on surgeons. A pilot study completed in Wales found that new consultants in particular were at risk of burnout.^6^ Questions regarding optimal methods to correct any significant stress present in the profession were felt necessary. It is believed that this is an issue that appears to be significantly more important in healthcare than is currently acknowledged. Many clinicians know individual surgeons affected by stress caused directly by taking part in cataract surgery or are affected by stress themselves.^6^ However, to date, there has been no attempt to explore the prevalence of such stress amongst practicing cataract surgeons. We believe this is even more significant, given the effect of the Covid-19 pandemic on elective surgery. A global expert response study estimated that 28 404 603 operations were cancelled or postponed simply during the peak 12 weeks of the first wave of Covid-19, and even if countries increased normal surgical volume by 20% it would take a median of 45 weeks to clear this backlog of operations.^7^ We now know the repercussions of the pandemic are far more extensive than this short window. It is estimated that waiting times have increased between 7–42% with a considerable increase in disease related morbidity and mortality.^8–11^

However, as services return normality within the near future, this has inevitably had a considerable impact on the mental well-being of the workforce who have dealt with this backlog. Thus, work supporting the understanding of factors that may predispose to clinician burnout would appear to be more important than ever before.

We aimed to evaluate the prevalence of stress-induced burnout among cataract surgeons in the United Kingdom by exploring demographic and personal factors. This would in turn allow us to propose potential solutions from these results to cope with the inevitable increased workload over the coming years, whilst ensuring the well-being of our national work force and optimising surgical outcomes.

## Methods

After completing a pilot study in Wales,^6^ a nationwide cross-sectional survey was undertaken via the Royal College of Ophthalmologists (RCOphth), between December 2019 and February 2020. All consultants, trainees, specialty doctors and associate specialists (SAS) were invited to participate; this was divided into three individual sub-sections. All contributors were asked to complete Part 1, 2 and 3a whereas Part 3b was intended for consultants only (full questionnaire Supplementary Material 1). The Health Research Authority (HRA) decision tool confirmed ethical approval was not required for this study.

### Survey structure

Part one assembled demographic details including: current position, including year of training or time spent as consultant, how many cataract operations completed, current deanery/geographical location, age, number of cataract lists undertaken each week, subspecialty of interest and average hours slept prior to surgery.

Part two employed the MBI questionnaire. This has previously been validated for use in healthcare.^12–14^ Very small modifications were made to some of the questions in order to direct the ophthalmologist to the cataract surgery component of their job as opposed to the whole of their working life (Supplementary Material 1). The questionnaire itself consists of 22 questions that are scored through a seven-point Likert scale that ranges from 0 to 6. It is split into its three sections A (EE), B (DP) and C (PA). Each sub-section is subsequently summed and categorised as high, moderate, or low. Scoring 27 or more on EE, 13 or more in DP or 31 or less on PA was deemed to be indicative of high-level burnout for each subscale. Total burnout was calculated through scores recording a ‘high-level burnout’ in section A and/or B. This is the most implemented categorisation of high-burnout when using the MBI questionnaire.^15^

Part three consisted of two sub-divisions. Section 3A: asked if the clinician were paid the same amount for a job plan that did not involve undertaking cataract surgery, would they be willing to accept this. Section 3B: was aimed at consultants only. Questions included whether they currently supervise trainees, if supervising trainee surgeons is more stressful than performing independent lists and if given the choice would the preference be to operate without trainees.

### Distribution and statistical analysis

Invitation to participate was disseminated via the RCOphth electronically to all registered members including trainees in the United Kingdom. Figures provided directly by the RCOphth and its published census estimate between 1800–2200 practicing cataract surgeons in the United Kingdom,^16^ of which 702 are trainees. A subsequent reminder was also delivered two weeks prior to the intended closing date. Anonymity was guaranteed through all stages of data collection. Information regarding gender, ethnicity and marital status was not collected.

Statistical analysis was completed through STATA 14.1 (STATA Corp, College Station, TX). Descriptive statistics were used to summarise demographic data as percentages and averages. Mann-Whitney U tests were used when comparing subsection scores between consultants and trainees. Multivariate logistic regression modelling was completed for individual domains. This evaluated separately the associations between ‘High-level burnout’ and employment status (consultant/ associate specialist or trainee), age, number of operations completed, lists per week and hours slept per night. Results were reported as odds ratios (OR) with 95 percent confidence intervals (95% CI). Statistical significance was set at *p *<* *0.05.

## Results

### Demographics

**Figure 1.**
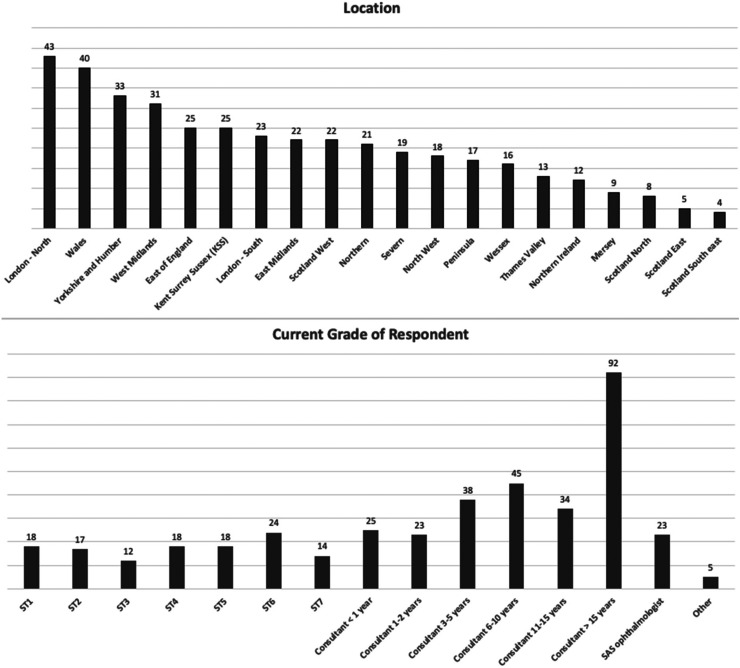
Demographic details of location and grade of respondents (number of total responses).

A total of 406 respondents completed the survey which was administered electronically via a web link. This consisted of 121 trainees (30%) and 285 consultants, SAS or other ophthalmologists (70%) with a crude response rate of 18%^16^ (17% of trainees) (5% margin of error for study responses^17^). By far the highest cohort of respondents were consultants practicing for more than 15 years (n* *=* *92) consisting of 23% of the total respondent population. Responses were gathered from all 20 respective deaneries within the United Kingdom. The highest response rates were seen in Wales (n* *=* *40) and London-North (n* *=* *43). The majority of the population was aged between 31–40 (n* *=* *128) and 51–60 (n* *=* *101). A diverse response was seen encompassing all the ophthalmic subspecialties, with the largest individual response 17% (n* *=* *70) from those currently working within medical retina. On average ophthalmologists slept 7 h prior to cataract surgery 49% (n* *=* *200) with the most common number of cataract lists being 1 per week 33% (n* *=* *137). Demographic details for location and grade of respondent can be seen in Figure 1:

### High-level burnout

**Figure 2.**
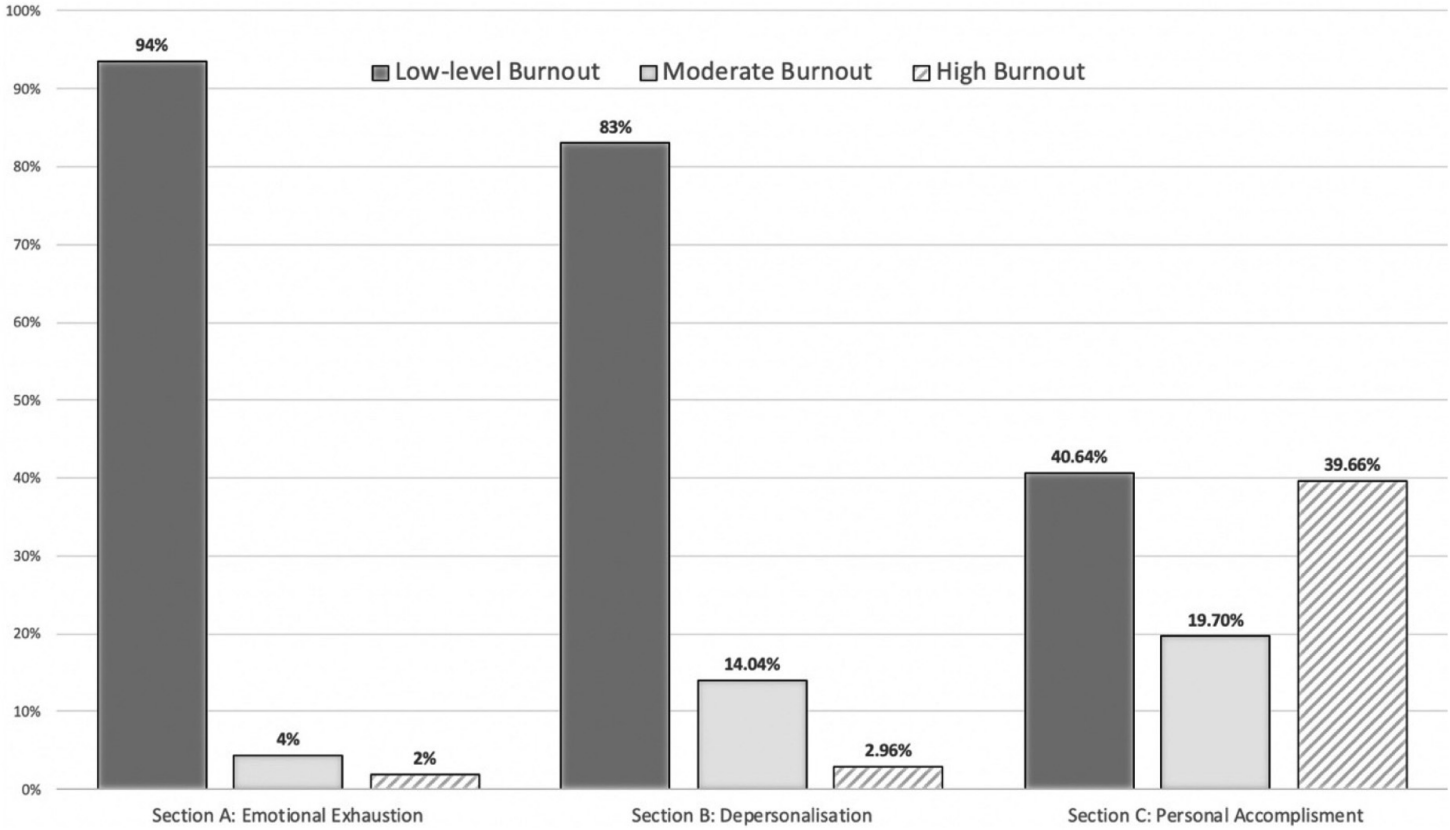
Percentage of correspondents indicating low, moderate and high burnout in each domain of the MBI questionnaire.

Section two looked to estimate stress-related burnout using the MBI questionnaire. Depending on individual subscale scoring, each section of this questionnaire provided us with information regarding levels of burnout individually. Section A (EE) found 1.97% of respondents reporting high and 4% moderate burnout respectively. Section B (DP) noted this to be 2.96% (high) and 14.04% (moderate). However, Incidence of high-level burnout was considerably higher in the PA domain (Section C) with 40% of the respondents displaying high burnout and 19.7% moderate burnout (Figure 2). The estimated total prevalence of high-level burnout in section A and/or B was calculated to be 3.45% (n* *=* *14). With 96% (n* *=* *135) of consultants >15 years and <2 years found to be either moderately or highly burnout. Evaluation of mean scores equating variance between consultants and trainees found no significant difference apart from PA (*p *<* *0.001) (Table 1).

**Table 1.** Mean scores for individual sections of the MBI scores between consultants and trainees for each domain of the MBI questionnaire.

Section A : Burnout (or depressive anxiety syndrome):	Mean score	SD	95% CI	*P*-value	
Consultant	6.62	7.16	5.79	7.46	0.502
Trainee	6.45	5.96	5.38	7.53	
Section B: “Depersonalization” (or loss of empathy):	Mean score	SD	95% CI	*P*-value	
Consultant	3.59	4.69	3.04	4.13	0.5945
Trainee	3.40	3.58	2.76	4.05	
Section C: The reduction of personal achievement	Mean score	SD	95% CI	*P*-value	
Consultant	35.68	10.18	34.50	36.87	<0.001
Trainee	32.26	9.68	30.51	34.00	

### Section C – personal achievement

Section C (PA) was subsequently further explored given the considerably high level of burnout identified.

A reduction in personal accomplishment would suggest that *‘The individual assesses himself negatively, feels he is unable to move the situation forward. This component represents the demotivating effects of a difficult, repetitive situation leading to failure despite efforts. The person begins to doubt his genuine abilities to accomplish things’*^18^

Within this cohort 69.23% of Cataract, 55.93% Vitreoretinal and 54.54% of Glaucoma specialists indicated the highest level of burnout within this domain. With the majority aged between 31–60 79% (n* *=* *87). Multivariate logistic regression modelling adjusting for age, training grade, number of cataract operations performed, lists per week and hours slept prior to surgery was performed. Results indicated multiple factors to be associated with increased risk of burnout including: increasing Age 61+ (Odds Ratio, OR 2.99, (95% Confidence Interval, (CI)) 1.02–8.78, *p *=* *0.05), Number of cataract operations completed: >3000 (OR 2.98 (95%CI 1.03–8.64), *p *=* *0.04), Lists per week: 2: OR 2.99 (95% CI 1.38–6.47) *p *<* *0.01, 2.5: OR 8.95 (95% CI 2.58–31.02) *p *<* *0.01, 3 or more: OR 2.64 (95% CI 1.07–6.54) *p *=* *0.042 (Table 2)

**Table 2.** Multivariate logistic regression modelling adjusted for age, training grade, number of cataract procedures completed, list per week and hours slept prior to surgery, evaluating high burnout in the PA domain.

	Odds ratio	95% confidence interval	*p*-value
*Age*			
20–30	0.932	0.29–3.04	0.908
31–40	0.967	0.42–2.18	0.935
41–50	Ref.		
51–60	0.67	0.34–1.30	0.236
61+	2.99	1.02–8.78	0.046
*Training grade*			
Trainee	Ref.		
Consultant Or SAS	1.12	0.49–2.55	0.788
*Number of Cataract procedures completed*			
<500	Ref.		
501–1500	1.78	0.77–4.08	0.174
1501–3000	2.67	0.98–7.31	0.055
>3000	2.978	1.03–8.64	0.044
*Lists per week*			
<1	Ref.		
1	1.44	0.70–2.96	0.326
1.5	1.33	0.54–3.26	0.526
2	2.99	1.38–6.47	0.005
2.5	8.95	2.58–31.02	0.001
3 or more	2.64	1.07–6.54	0.036
*Hours Slept prior to surgery*			
4	0.85	0.09–8.18	0.890
5	0.64	0.24–1.69	0.367
6	1.02	0.59–1.75	0.945
7	Ref.		
8	0.52	0.28–0.96	0.038
>8	2.72	0.42–17.50	0.291

Footnote: SAS: Sp**e**cialty and Associate Specialist doctor, Ref: Reference, PA: Personal Accomplishment.

### Part 3A

A significant proportion of respondents, 17% (n* *=* *69) stated that they would be willing to give up their surgical duties if they were paid the same. Of those willing to relinquish surgical responsibilities, proportionally calculated against numbers from their respective specialties, the largest cohort consisted of respondents from medical retina 29% (n* *=* *20) followed by paediatrics 28% (n* *=* *11) and oculoplastics 24% (n* *=* *11). Additional information on training grade highlighted that this consisted predominately of new consultants 1–2 years after CCT, or consultants with more than 15 years of experience. Most of these surgeons had completed either 701–1500 or >6000 cataract procedures each (20% (n* *=* *14)) respectively.

### Part 3B

**Figure 3.**
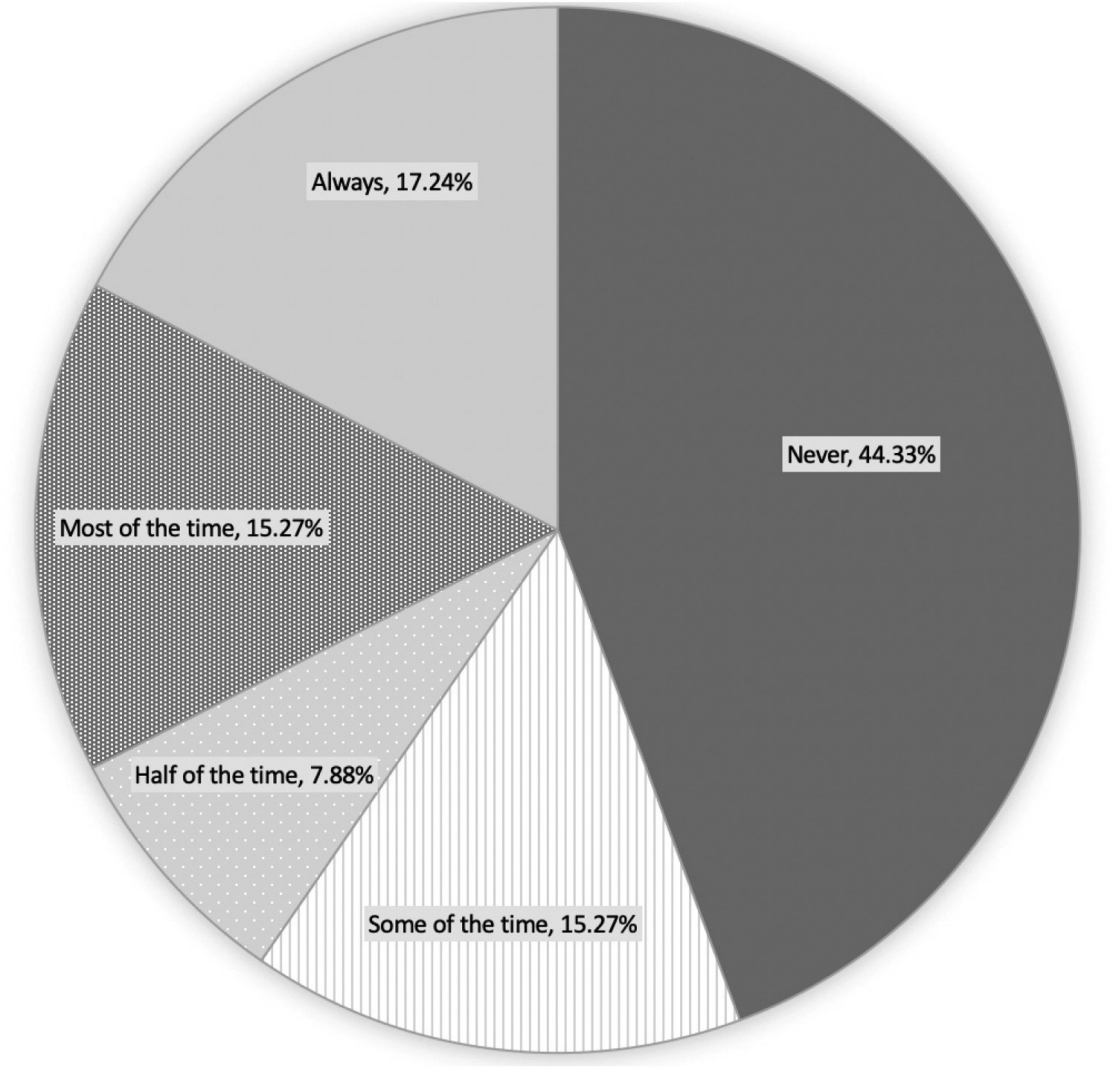
Consultant opinions on whether supervising trainee cataract surgeons is more stressful than performing independent lists.

Of the 285 consultants that participated 224 indicated that they supervised trainees. 55% of these consultants indicated that they find supervising trainees stressful to varying degrees, with 44% indicating that they would prefer to operate without trainees on their list. The level of stress indicated can be seen in Figure 3.

## Discussion

Our survey response rate of 18% compares significantly well to other nationally distributed surveys.^19–21^ We found the ‘high-level burnout’ rate linked with cataract surgery in the United Kingdom to be 3.45%, a figure predominately comprising of Consultant or SAS Ophthalmologists. This however does not **describe** the 40% high-burnout rate identified in PA. Burnout (moderate to high) and those willing to give up surgical responsibilities appears to be more significant amongst ophthalmologists newly appointed as consultants, those further on in their respective careers and individuals who perform less than one cataract list per week. When we considered individual sub-section scores, high-level of burnout appears to be most prevailing within the domain of personal accomplishment (PA). This appears to be more dominant amongst sub-specialists working in glaucoma and vitreoretinal surgery. Our modelling suggested a detrimental effect of increasing lists and subsequently total cataracts performed and its impact on clinician personal achievement and mental well-being. Conversely, getting a good night's sleep appeared to be protective. We identified a significant proportion of consultants willing to give up surgical responsibilities, however we cannot say this is simply due to finding operating stressful and requires further evaluation. Lastly, we discovered a high proportion of consultants find supervising trainees stressful with an even higher fraction favouring to operate without a trainee on their list altogether.

The three key dimensions of burnout consist of an overwhelming mental and physical fatigue, feeling of detachment from the job and a sense of ineffectiveness with associated poor work satisfaction.^22,23^ It appears to be a multifactorial problem stemming from high patient volume, financial pressures, long working hours and inefficiencies posed by poor work-life balance.^24^ These have been linked with the evolution of modern-day medicine; be it the regulatory burden, operational policies or institutional inefficiencies.^25^ The increasingly litigious environment and high patient volumes have further increased the burden of care and influenced the manner in which we practice.^26^ Burnout itself has been linked with an increased risk of depression, anxiety, sleep disturbance, substance misuse and even marital discord.^27^ Unfortunately, this impacts not only the clinicians in question but also the patients they care for.

Literature would suggest burnout to be an increasing problem, with prevalence levels reported to be between 18.7 to 74.8% worldwide.^15,28^ One prospective cohort study of 3588 s year resident (trainee) physicians in the United States reported burnout occurring in 45.2% of participants with a career choice regret of 14.1%.^29^ There were significant disparities in prevalence by clinical specialty ranging between 29.6%-63.8% for symptoms linked with burnout. Interestingly, levels of burnout appeared to be more commonly seen in females, ethnic minorities, and those not in relationships. Authors of this study estimated ophthalmology related burnout to be 55.8%.^29^ A recently published systematic review noted a worldwide aggregate prevalence of physician burnout to be 51%, with occurrence particularly high in neurology, radiology and general surgery.^15,30^ It highlighted that burnout among trainees was under recognised. Subgroup analysis looking at ophthalmology as a specialty could not be completed due to inadequate number of studies with only a few authors having attempted to evaluate its true prevalence.^31–33^ To date no one has evaluated how the world's most common surgical procedure, cataract surgery, has impacted the mental health of its workforce.

Given the nature of work and exposure to high-pressured environments, the vocation of medicine has frequently been reported to be associated with varying levels of stress to those involved.^34^ Doctors are known to be at a higher risk of anxiety, depression and even substance abuse in comparison to the wider population.^35^ More recently elements such as financial constraints, litigious fear and administrative limitations have been reported to be linked with burnout.^36–38^ It is a combination of factors which can have an adverse effect on both the physical and mental well-being of physicians. Thus, the concept of resilience and adoption of coping strategies has gained increasing importance over the last decade.^39^ Frequently reported coping strategies often employed by doctors appear to vary depending on personal, social and environmental factors. Some believe emotional support to be crucial, a concept that involves humour, religion, and instrumental support of those around them.^40^ Others utilise forms of adaptive coping strategies which may involve taking a break/pausing prior to reacting, or even physical activity such as going for a run.^41^ Conversely some physicians report planning to be vital, incorporating elements of positive reframing and acceptance when dealing with stressful situations that may lead to burnout. However, there remains a cohort of individuals that adopt harmful maladaptive coping strategies such as denial, self-blame, self-distraction and in extreme cases substance abuse.^42^ It is therefore essential to educate clinicians and establish practice which mitigates burnout.

Stress induced by supervising trainees will inevitably vary depending on the seniority and competence of the trainee.^43,44^ This survey indicates increased stress-related burnout amongst new consultants, very senior consultants and people working in certain subspecialties such as medical retina. Almost a fifth of respondents would give up cataract surgery if they could and a large number would prefer to operate without trainees. Our results indicate that new consultants who were at higher risk of burn out may require supportive mentoring on taking up their consultant posts; likewise for consultants at the end of their career with high burn out. The profession as a whole can benefit from policies implemented around the world including self-care workshops, mindfulness-based interventions and encouragement to exercise/improve work-life balance, all of which have been shown to improve individual aspects of burnout^45^

Surgery undertaken in the training period is associated with a higher complication rate and most consultants are obliged to teach and supervise trainees allocated to their firms, to learn cataract surgery. The finding that some consultants find this supervision stressful; as the outcome and care remains their responsibility, rather than the trainee may explain the differential rate of high burn out. The fact that almost a fifth of ophthalmologists would give up cataract surgery given the choice might suggest the need of developing a curriculum for a workforce, where surgery is not a priority.

Whilst it is essential that strategies are adopted to help prevent or reduce burnout on both an individual and institutional level there might be other solutions. The Identification of ophthalmologists who would prefer not to operate at the beginning of their careers would prevent much anguish during training and their consultant career if an alternative training pathway without cataract surgery supported by an appropriate curriculum is developed by regulatory bodies. Medical ophthalmology has a separate training programme, however the current ophthalmology training programmes which mandate competency in cataract surgery may be associated with the negative impacts of anxiety during cataract surgery and poor job satisfaction. Development of a workforce which includes ophthalmologists who do not undertake cataract surgery and those who undertake high volume cataract surgery to suit the needs of the population needs may promote the well-being of the ophthalmologists of the future. With this increase in ‘super specialists’, we hope would reduce the burden on current clinicians solely specialising in cataract surgery. Ophthalmologists from certain specialties, such as medical retina, are at higher risk of moderate to high burnout due to cataract surgery, it is a belief that such subspecialties could potentially be thought of as non-operating fields in the future. Additionally, it appears that a significant proportion of Ophthalmologists have lost a sense of personal accomplishment with cataract surgery, particularly those working in cataracts, glaucoma and vitreoretinal Surgery. Medical professionals in general are critical of themselves when things go wrong, and rarely celebrate routine success or achievements. Perhaps methods of regular recognition should be adopted, rewarding those who have continued to serve and dedicate their skills to the profession and society.

Optimal methods of evaluating burnout can be difficult to decide. Many have argued whether each dimension should be evaluated and reported separately or combined. On conceptual grounds it is preferable to treat burnout as a multidimensional construct, however when estimating its prevalence in research it is significantly easier to treat this as a unidimensional variable. The MBI questionnaire is by far the most utilised tool to evaluate this.^46^ Although, there appears to be variability in scoring methods, we employed the most validated techniques described in literature. Our ability to reach a wide representation of practicing ophthalmologists within the United Kingdom ensured the elimination of any location bias. We completed a pilot study within Wales prior to nationwide dissemination,^6^ establishing the need for this with our results uncovering key factors which may influence the impact of stress on our lives.

Future directions of research should include the effect of well-being interventions on the level of burn out for young consultants. Detailed work on subspecialty representation and the burnout levels may help to support designing a curriculum for training ophthalmologists without cataract surgery.

When critically evaluating our methods, we noted several limitations to our study. Our response rate could have been higher. The limited number of responses advises caution in generalising our results to the entire population of UK ophthalmologists. The MBI questionnaire itself was developed as a research tool to assist the understanding of burnout, and although frequently used there remains significant heterogeneity in its interpretation. This leads to less reliable results. Despite using a validated inventory, response bias and participation bias cannot be eliminated from any self-reported surveys and may affect true representation. Although commonly associated with burnout, we decided not to evaluate differences in sex, ethnicity and family status as we felt these demographic details did not align with our intended objectives. Additionally, given the small proportions of ophthalmologists indicating high-level burnout in section A and/or B, we deemed further description of these results were not appropriate. This limited the methods by which we could best summarise the relationships between key variables.

## Conclusions

The prevalence of stress induced burnout by cataract surgery appears to be low overall but significant amongst certain groups and domains. There has been an unfortunate reduction in the feeling of personal accomplishment within the discipline. A significant proportion of ophthalmologists appear to be willing to give up surgical responsibilities, influenced by varying degrees of burnout. More must be done to further evaluate the impact of burnout on the specialty across clinical and surgical responsibilities with the inevitable increased burden on services.

## Supplemental Material

sj-docx-1-ejo-10.1177_11206721231154611 - Supplemental material for Stress and cataract surgery: A nationwide study evaluating surgeon burnout Click here for additional data file. Supplemental material, sj-docx-1-ejo-10.1177_11206721231154611 for Stress and cataract surgery: A nationwide study evaluating surgeon burnout by Abdus Samad Ansari, Annie See Wah Tung, David M Wright, Patrick Watts and Gwyn Samuel Williams in European Journal of Ophthalmology
