# Supplementary material for: Stress and cataract surgery: A nationwide study evaluating surgeon burnout
Source: Eur J Ophthalmol. 2023 Feb 3;33(4):1640–9. doi: 10.1177/11206721231154611 (PMC10331514; doi:10.1177/11206721231154611)
Supplement: sj-docx-1-ejo-10.1177_11206721231154611 - Supplemental material for Stress and cataract surgery: A nationwide study evaluating surgeon burnout [file sj-docx-1-ejo-10.1177_11206721231154611.docx]

**Examining the Perceived Anxiety of Undertaking Phacoemulsification Surgery**

*Thank you for participating in our survey examining how stressful you find phacoemulsification cataract surgery.*

*This is an issue that appears to be significantly more important to the health of our profession than is currently acknowledged and whilst many of us know individual surgeons affected by stress caused directly by taking part in cataract surgery, or are affected by stress ourselves, there has been no attempt thus far to explore the prevalence of such stress amongst the profession as a whole.*

*Your answers will help us explore a possible major stressor in the lives of many ophthalmologists. This will in turn I hope help us as a profession to develop robust methods of helping the cataract surgeons of the present, as well as the future. Results from the survey dataset will be anonymised and all findings will be reported in an aggregated form*

**Section 1**

**Your Background**

Which of the following best describes you?

1. *ST1*
2. *ST2*
3. *ST3*
4. *ST4*
5. *ST5*
6. *ST6*
7. *ST7*
8. *Consultant < 1 year*
9. *Consultant 1-2 years*
10. *Consultant 3-5 years*
11. *Consultant 6-10 years*
12. *Consultant 11-15 years*
13. *Consultant > 15 years*
14. *SAS ophthalmologist*
15. *Other*

How many Cataract operations have you approximately completed?

Deanery

1. *London - North*
2. *Wales*
3. *Yorkshire and Humber*
4. *West Midlands*
5. *East of England*
6. *Kent Surrey Sussex*
7. *London - South*
8. *East Midlands*
9. *Scotland West*
10. *Northern*
11. *Severn*
12. *North West*
13. *Peninsula*
14. *Wessex*
15. *Thames Valley*
16. *Northern Ireland*
17. *Mersey*
18. *Scotland North*
19. *Scotland East*
20. *Scotland South east*

Age

1. *20-30*
2. *31-40*
3. *41-50*
4. *51-60*
5. *60+*

How many CATARACT Operating lists on average do you undertake each week?

1. <1
2. 1
3. 1.5
4. 2
5. 2.5
6. 3 or more

What is your subspecialty interest?

1. *Medical Retina*
2. *Vitreoretinal*
3. *Glaucoma*
4. *Cornea*
5. *Oculoplastic*
6. *Paediatrics*
7. *General*
8. *Cataract*
9. *Neuro-Ophthalmology*
10. *Emergency Ophthalmology*
11. *Not Decided*

If other subspecialty of interest:

Average hours slept prior to surgery

1. <3
2. 3
3. 4
4. 5
5. 6
6. **7**
7. 8
8. >8

**Section 2**

**Evaluating Stress**

The following questions simply aid in evaluating a Cataract surgeons risk of burn out.

Please answer all the questions

**Section A (Emotional Exhaustion)**

1) I feel emotionally drained/exhausted after performing cataract surgery

- Never
- A few times per year
- Once a month
- A few times per month
- Once a week
- A few times per week
- Every day

2) Working with theatre staff all day requires a great deal of effort

- Never
- A few times per year
- Once a month
- A few times per month
- Once a week
- A few times per week
- Every day

3) I feel like cataract surgery is breaking me down

- Never
- A few times per year
- Once a month
- A few times per month
- Once a week
- A few times per week
- Every day

4) I feel frustrated by cataract surgery

- Never
- A few times per year
- Once a month
- A few times per month
- Once a week
- A few times per week
- Every day

5) I feel I work too hard whilst operating

- Never
- A few times per year
- Once a month
- A few times per month
- Once a week
- A few times per week
- Every day

6) Interacting with theatre staff directly puts too much stress on me

- Never
- A few times per year
- Once a month
- A few times per month
- Once a week
- A few times per week
- Every day

7) I feel like I am at the end of my rope

- Never
- A few times per year
- Once a month
- A few times per month
- Once a week
- A few times per week
- Every day

**Section B (Depersonalisation)**

8) I feel that I treat some patients as if they were impersonal objects

- Never
- A few times per year
- Once a month
- A few times per month
- Once a week
- A few times per week
- Every day

9) I feel tired in the morning when I get up and have to face another day operating

- Never
- A few times per year
- Once a month
- A few times per month
- Once a week
- A few times per week
- Every day

10) I feel that some patients blame me for their problems after cataract surgery

- Never
- A few times per year
- Once a month
- A few times per month
- Once a week
- A few times per week
- Every day

11) I feel like I am at the end of my patience at the end of a theatre list

- Never
- A few times per year
- Once a month
- A few times per month
- Once a week
- A few times per week
- Every day

12) I don’t really care what happens to some patients during cataract surgery

- Never
- A few times per year
- Once a month
- A few times per month
- Once a week
- A few times per week
- Every day

13) I have become more uncaring towards people since I have started operating

- Never
- A few times per year
- Once a month
- A few times per month
- Once a week
- A few times per week
- Every day

14) I worry that cataract surgery is hardening me emotionally

- Never
- A few times per year
- Once a month
- A few times per month
- Once a week
- A few times per week
- Every day

**Section C (Personal Accomplishment)**

15) I have accomplished many worthwhile things whilst performing cataract surgery

- Never
- A few times per year
- Once a month
- A few times per month
- Once a week
- A few times per week
- Every day

16) I feel very energetic whilst in theatre

- Never
- A few times per year
- Once a month
- A few times per month
- Once a week
- A few times per week
- Every day

17) I can easily understand how my patients feel about undergoing cataract surgery

- Never
- A few times per year
- Once a month
- A few times per month
- Once a week
- A few times per week
- Every day

18) I deal very effectively with the problems of my patients undergoing cataract surgery

- Never
- A few times per year
- Once a month
- A few times per month
- Once a week
- A few times per week
- Every day

19) Whilst operating, I deal with emotional problems very calmly

- Never
- A few times per year
- Once a month
- A few times per month
- Once a week
- A few times per week
- Every day

20) I feel that I am making a positive influence on other peoples lives through cataract surgery

- Never
- A few times per year
- Once a month
- A few times per month
- Once a week
- A few times per week
- Every day

21) I can easily create a relaxed atmosphere with my patients whilst performing cataract surgery

- Never
- A few times per year
- Once a month
- A few times per month
- Once a week
- A few times per week
- Every day

22) I feel refreshed after working closely with my patients

- Never
- A few times per year
- Once a month
- A few times per month
- Once a week
- A few times per week
- Every day

**Section 3A**

If you were paid the same amount for a job plan that did not involve undertaking cataract surgery would you take it?"

- Yes
- No

**Section 3B**

*The following questions are for Consultants who Supervise trainees*

Are you a consultant who supervises trainees?

- Yes – Please continue
- No- Thank you, this section does not require completing

Do you find supervising trainee cataract surgeons more stressful than performing independent lists?

- Never
- Some of the time
- Half of the time
- Most of the Time
- Always

2) If you had a choice would you prefer to operate without trainees on your list?

- Yes
- No

Thank you for completing this questionnaire
